# Stromal interaction molecule 1 haploinsufficiency causes maladaptive response to pressure overload

**DOI:** 10.1371/journal.pone.0187950

**Published:** 2017-11-16

**Authors:** Takayoshi Ohba, Hiroyuki Watanabe, Manabu Murakami, Kenji Iino, Takeshi Adachi, Yoshihiro Baba, Tomohiro Kurosaki, Kyoichi Ono, Hiroshi Ito

**Affiliations:** 1 Department of Cell Physiology, Akita University Graduate School of Medicine, Akita, Japan; 2 Department of Cardiovascular and Respiratory Medicine, Akita University Graduate School of Medicine, Akita, Japan; 3 Department of Pharmacology, Hirosaki University, Graduate School of Medicine, Aomori, Japan; 4 Laboratory for Lymphocyte Differentiation, WPI Immunology Frontier Research Center, Osaka University, Osaka, Japan; Texas A&M University Health Sciences Center, UNITED STATES

## Abstract

Stromal interaction molecule 1 (STIM1), an endo/sarcoplasmic reticulum Ca^2+^ sensor, has been shown to control a Ca^2+^-dependent signal that promotes cardiac hypertrophy. However, whether STIM1 has adaptive role that helps to protect against cardiac overload stress remains unknown. We hypothesized that STIM1 deficiency causes a maladaptive response to pressure overload stress. We investigated STIM1 heterozygous KO (*STIM1*^+/–^) mice hearts, in which STIM1 protein levels decreased to 27% of wild-type (WT) with no compensatory increase in STIM2. Under stress-free conditions, no significant differences were observed in electrocardiographic and echocardiographic parameters or blood pressure between *STIM1*^+/–^and WT mice. However, when *STIM1*^*+/–*^mice were subjected to transverse aortic constriction (TAC), *STIM1*^*+/–*^mice had a higher mortality rate than WT mice. The TAC-induced increase in the heart weight to body weight ratio (mean mg/g ± standard error of the mean) was significantly inhibited in *STIM1*^+/–^mice (WT sham, 4.12 ± 0.14; WT TAC, 6.23 ± 0.40; *STIM1*^+/–^sham, 4.53 ± 0.16; *STIM1*^+/–^TAC, 4.63 ± 0.08). Reverse transcription-polymerase chain reaction analysis of the left ventricles of TAC-treated *STIM1*^+/–^mice showed inhibited induction of cardiac fetal genes, including those encoding brain and atrial natriuretic proteins. Western blot analysis showed upregulated expression of transient receptor potential channel 1 (TRPC1) in TAC-treated WT mice, but suppressed expression in TAC-treated *STIM1*^+/–^mice. Taken together, the hearts of STIM1 haploinsufficient mice had a superficial resemblance to the WT phenotype under stress-free conditions; however, STIM1 haploinsufficient mice showed a maladaptive response to cardiac pressure overload.

## Introduction

The heart is capable of remodeling in response to environmental stressors. A variety of cardiovascular diseases can cause pathological hypertrophic growth, which entails the genetic reprogramming of fetal cardiac genes resulting in increased protein synthesis and cell size within individual myocytes. Increased intracellular Ca^2+^ underlies cardiomyocyte hypertrophy through Ca^2+^-dependent signals. Accumulating evidence suggests that the response to hypertrophic signals involves Ca^2+^ entry channels, including canonical transient receptor potential channels (TRPCs) and Orai channels, as well as stromal interaction molecule 1 (STIM1), an endo/sarcoplasmic reticulum Ca^2+^ sensor [1–10].

STIM1 is highly conserved and appears to be ubiquitous in all eukaryotic cells [11]. STIM1 was initially shown to act as a Ca^2+^ sensor in the endoplasmic reticulum (ER) and to mediate store-operated Ca^2+^ entry (SOCE), a major mechanism of Ca^2+^ entry in nearly all non-excitable cells. Upon depletion of Ca^2+^ in the ER, STIM1 relocalizes within the ER to regions that are in close vicinity to the cytoplasmic membrane, and then promotes the opening of ORAI1 in the plasma membrane to induce the entry of extracellular Ca^2+^, resulting in transcriptional activation through nuclear factor of activated T cells (NFAT) [12]. In addition, STIM1 has also been reported to interact with TRPC family in various cell types [9, 10, 13]. Indeed, our *in vitro* study showed that STIM1 knockdown inhibited the upregulation of TRPC1 and led to the abrogation of SOCE and a robust decrease in the activation of NFAT, which resulted in the inhibition of cardiomyocyte hypertrophy [7]. Likewise, two other groups reported that STIM1 knockdown induced loss of SOCE and suppressed agonist-triggered cardiac hypertrophy in primary cultured neonatal cardiomyocytes [8, 10]. In humans, STIM1 loss-of-function mutations were identified in patients afflicted with an immunodeficiency and autoimmunity syndrome. The STIM1 loss-of-function mutation abrogates SOCE and impairs lymphocyte activation, mainly due to a failure to activate the calcineurin/NFAT system [14]. STIM1 deficiency has been reported in ~20 patients [15–19]; interestingly, almost every patient manifests myopathy as well as immunodeficiency. Therefore, it is reasonable to speculate that STIM1 deficiency might lead to heart disease in humans. However, the STIM1-deficient patients had no clinical features of heart disease. One possible explanation is that STIM1-deficient patients have early mortality without clinically important cardiac hypertrophy that developed over an extended period of time. We hypothesized that STIM1 deficiency may cause a maladaptive response to increased load. Ubiquitous germ-line deletion of STIM1 is embryonically lethal [20], and therefore we used STIM1 haploinsufficiency (*STIM1*^*+/–*^) mice to address this issue.

In the present study, the cardiac phenotype of *STIM1*^*+/–*^mice was examined, and the mice were subjected to transverse aortic constriction (TAC) to determine the adaptive role of STIM1 under pressure-overload conditions.

## Materials and methods

### 1. STIM1 hetero-knockout (KO) (*STIM1*^*+/–*^) mice

The generation of *STIM1*^*+/–*^mice using the Cre/loxP system has been described previously [20]. This study conformed to the Akita University Graduate School of Medicine principles for animal care and with the US National Institutes of Health Guide for the Care and Use of Laboratory Animals (NIH Publication No. 85–23, revised 1996). Although we considered humane endpoints, we could not use them because of unexpected deaths. 14 mice died without euthanasia, which exhibited no clinical symptoms prior to their death. The Animal Ethics Committee of the Akita University School of Medicine has approved all animal care, experiments, and methods including survival study with any unexpected deaths. For the survival study, humane endpoints criteria is rapid or progressive weight loss of more than 20% of the body weight, dehydration determined by an increase in skin tenting, sunken eyes, respiratory symptoms such as labored breathing, nasal discharge, coughing, or cyanosis. The animals were carefully monitored three times a day.

### 2. Blood pressure (BP) measurements

BP and heart rate (HR) were measured in mice at 10 weeks of age using a tail-cuff system (BP-98A; Softron, Tokyo, Japan) without anesthetic.

### 3. Echocardiography

Echocardiography (echo) with concurrent electrocardiogram (ECG) was performed as described previously [21]. Briefly, mice were anesthetized with isoflurane (1%) in O_2_, and an echo was performed using a Vevo770 equipped with a 20–60 MHz mechanical transducer. Three to 5 beats were averaged for each measurement. The heart was first imaged in two-dimensional (2D) mode in the parasternal short-axis view. From this view, an M-mode cursor was positioned perpendicular to the interventricular septum and posterior wall of the left ventricle (LV) at the level of the chordae tendineae. From this position, M-mode images were obtained to measure wall thickness and chamber dimensions using the leading-edge convention adapted by the American Society of Echocardiography.

### 4. Minimally invasive TAC procedure

Minimally invasive aortic constriction is a modification of 4 techniques described previously [22–25]. For the banding procedure, 10-week-old male mice were anesthetized with a mixture of ketamine (90 mg/kg body weight [BW] administered intraperitoneally) and xylazine (10 mg/kg BW intraperitoneally), and TAC was performed through an upper partial sternotomy (0.2–0.3 cm) in spontaneously breathing nonintubated mice. Particular care was taken not to touch or damage the parietal pleura, preventing pneumothorax development. The aortic arch was carefully exposed, and a 7–0 silk suture was used to tightly constrict the aorta between the brachiocephalic trunk and the left carotid artery over a 0.3 mm wire. Once the suture was tied, the wire was removed. Sham-operated animals were subjected to the same procedure without aortic banding. The animals were sacrificed and the hearts were isolated 4 weeks after the operation. The constriction rate of the aorta was calculated, and a rate greater than 94% was considered a success. For histological analysis, 5-μm-thick sections were cut and stained with hematoxylin and eosin (H&E). The survival rate of *STIM1*^*+/–*^and wild-type (WT) mice after the TAC or sham surgery was analyzed by the Kaplan-Meier method and the log-rank test.

### 5. Reverse transcription-polymerase chain reaction (RT-PCR)

Total RNA was isolated from the LV of mice using ISOGEN II reagent (Nippon Gene, Tokyo, Japan). The RT reaction was performed using standard methods. For RT-PCR analysis, 2 μg of template was reverse-transcribed using oligo dT primer in a final volume of 20 μL. Primer sequences are provided in the S1 Table. Comparative RT-PCR was performed under the same conditions with 30 cycles; however, 25 cycles were used for reactions involving brain natriuretic protein (BNP), atrial natriuretic protein (ANF), and β-actin.

### 6. Western blotting

All protein samples were extracted from mice hearts. Protein concentrations were determined using a Bradford assay with bovine serum albumin (BSA) as a standard. To determine the protein expression of STIM1 (BD Biosciences, CA, USA), STIM2 (Cell Signaling Technology, MA, USA), TRPC1, TRPC3, TRPC4, and TRPC6 (Alomone Labs, Jerusalem, Israel), pNFATc4 (rabbit anti-mouse polyclonal antibody raised against short amino acid sequence containing Ser 168 and 170 dually phosphorylated NFATc4 of human origin, Santa Cruz Biotechnology, TX, USA), NFATc4 (rabbit anti-mouse polyclonal antibody raised against amino acids 125–198 of NFATc4 human origin, Santa Cruz Biotechnology, TX, USA), samples (50 μg) were run on 6% sodium dodecyl sulfate polyacrylamide gel electrophoresis (SDS-PAGE), transferred to polyvinylidene fluoride (PVDF) membranes, and rocked at 4°C for 1 h in blocking buffer (0.1% Tween 20 and 1% BSA in Tris-buffered saline). An enhanced chemiluminescence (ECL)-detection system was used to detect the bound antibodies. An anti-glyceraldehyde-3-phosphate dehydrogenase (GAPDH) monoclonal antibody (Santa Cruz Biotechnology, TX, USA) was used as an internal loading control.

### 7. Immunostaining

Heart tissues were fixed in 4% (v/v) paraformaldehyde in 0.1 M sodium phosphate buffer (pH 7.2) overnight at 4°C, embedded in paraffin wax, and cut into 3-μm-thick sections. For histological detection of NFATc4, the sections were incubated with the primary antibody against NFATc4 (rabbit anti-mouse polyclonal antibody raised against amino acids 125–198 of NFATc4 of human origin, Santa Cruz Biotechnology, TX, USA) at a 1:100 dilution at 4°C overnight. Then the slices were incubated with secondary antibody (Sigma-Aldrich, MO, USA) at a 1:160 dilution for 60 min, followed by incubation with DAPI for 30 min before observation. The nuclei appeared blue and the NFAT proteins appeared green under an inverted Zeiss LSM510META (Carl Zeiss, Oberkochen, Germany) confocal laser scanning microscope.

### 8. Statistical analysis

The data are presented as mean ± standard error of the mean (SEM). Differences were evaluated using unpaired Student’s *t*-tests for 2 groups. The quantitative data for 4groups were analyzed using a 2-way analysis of variance (ANOVA) followed by a Bonferroni post hoc test. Densitometry of Western blots and quantification of cross sectional area were performed by using NIH ImageJ software. A *p*-value < 0.05 was considered to indicate statistical significance.

## Results

### 1. Expression of STIM subtypes

**Fig 1:**
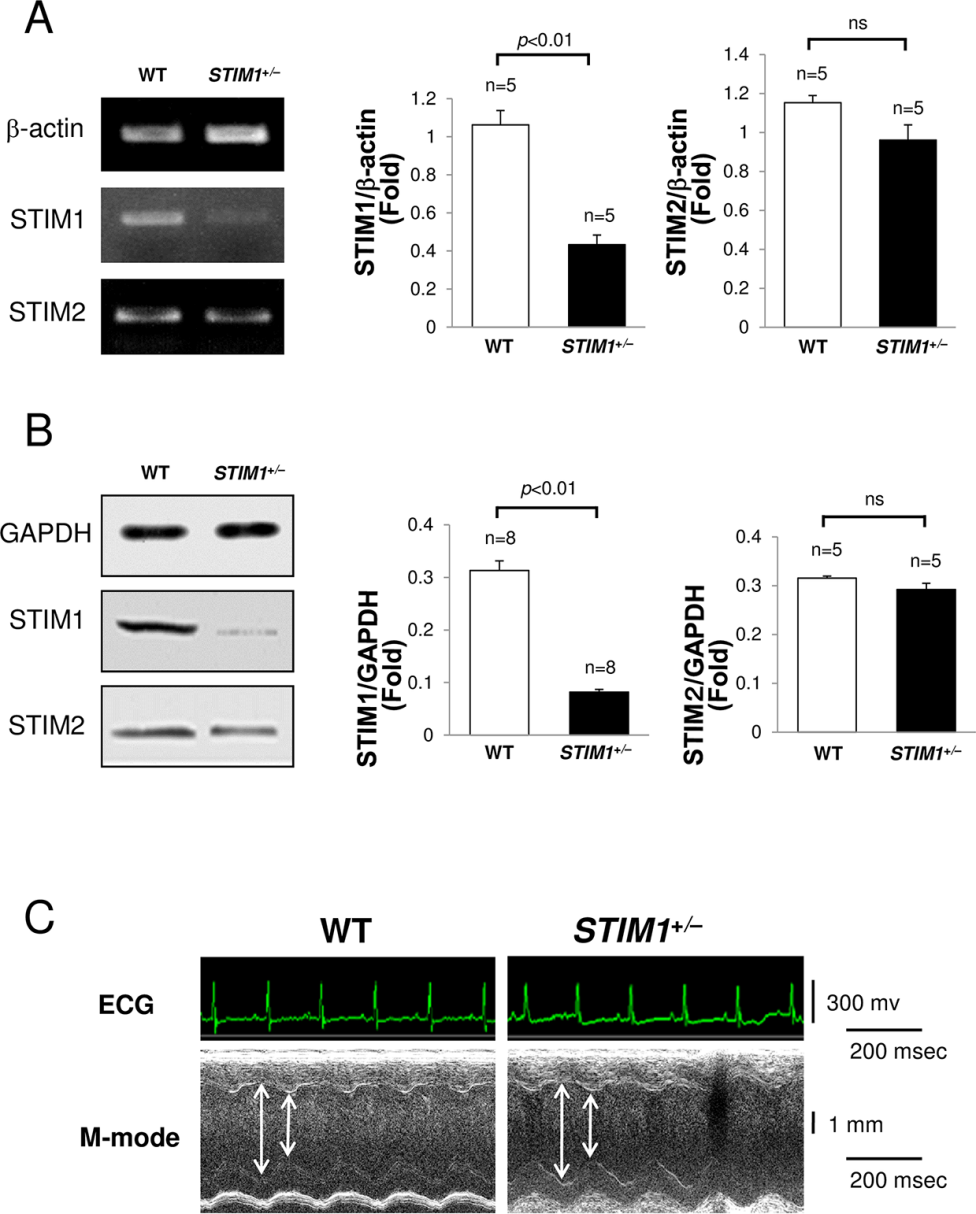
Analysis of the mice hearts. Analysis of the hearts of male mice heterozygous for stromal interaction molecule (STIM)1 and wild-type (WT) littermates (*STIM1*^+/–^versus *STIM1*^+/+^) at 10 weeks of age. (A) Comparative reverse transcription-polymerase chain reaction (RT-PCR) analysis of STIM1 and STIM2 gene expression. Each experiment was repeated 4 or more times with the same result. (B) Immunoblotting for STIM1 and STIM2. Each experiment was repeated 5 or more times with the same result. All values are means ± standard error of the mean (SEM). (C) Representative M-mode echocardiography with concurrent electrocardiogram in *STIM1*^+/–^and WT mice anesthetized with isoflurane (1%).

*STIM1*^+/–^male mice at 10 weeks of age were used in this study because the homozygous STIM1 global KO is neonatal lethal. *STIM1*^+/+^ WT littermates were used as controls. The levels of STIM1 and STIM2 are shown in Fig 1. The mRNA (Fig 1A) and protein levels (Fig 1B) of STIM1 in the LV of *STIM1*^+/–^mice decreased to approximately 42% and 27% of that of the WT, respectively, indicating STIM1 haploinsufficiency in the hearts of *STIM1*^+/–^mice. On the other hand, there were no significant differences in STIM2 subtypes expression between *STIM1*^+/–^and WT mice (Fig 1A and 1B), suggesting no compensatory increase in STIM2 in *STIM1*^+/–^hearts.

### 2. Phenotypes of STIM1-deficient mice

To investigate the cardiac phenotypes of *STIM1*^+/–^mice, heart weight (HW), BW, BP, and HR of *STIM1*^+/–^(n = 23) and littermate *STIM1*^+/+^ WT (n = 19) male mice were examined at 10 weeks of age. Although there were no differences in HW, a slight reduction was seen in the BW of *STIM1*^+/–^mice, consistent with previous studies [26, 27]. STIM1-deficient mice were smaller than WT mice due to a disorder of skeletal muscle development. The HW/BW ratio (mg/g) of *STIM1*^+/–^mice was increased slightly but not significantly. Systolic BP, diastolic BP, and HR were measured using a tail-cuff system without anesthetic agents. Systolic and diastolic BPs were reduced slightly in *STIM1*^+/–^mice compared with WT mice, but the differences were not significant (Table 1). Fig 1C, Table 2 and Table 3 contain representative ECG and M-mode echo data from 10-week-old *STIM1*^+/–^and WT mice anesthetized with isoflurane (1%). The ECG demonstrated no arrhythmias and no differences in ECG parameters between *STIM1*^+/–^and WT mice. The LV ejection fraction (EF), LV wall thickness, end diastolic volume, and LV mass determined by echo were unaffected, suggesting normal heart development and preserved cardiac contractile function in *STIM1*^+/–^mice at 10 weeks of age.

**Table 1:** Mice data at 10 weeks of age.

	n	HW (mg)	BW (mg)	HW/BW (mg/g)	Systolic BP (mmHg)	Diastolic BP (mmHg)	HR (bpm)
**WT**	23	122.9 ± 3.2	29.6 ± 3.4	4.17 ± 0.13	115.3 ± 4.0	76.5 ± 1.7	522.9 ± 10.3
***STIM+/-***	19	120.7 ± 2.4	26.6 ± 3.7	4.57 ± 0.13	113.2 ± 0.13	68.4 ± 8.5	528.5 ± 29.5
***p***		0.65	0.17	0.073	0.99	0.62	0.60

Heart weight (HW; mg), body weight (BW; g), systolic blood pressure (BP), diastolic BP, and heart rate (HR) are shown. BP and HR were measured using a tail-cuff system (BP-98A; Softron, Tokyo, Japan) without anesthetic. Statistical data are presented as means ± SEM.

**Table 2:** Electrocardiogram with concurrent echocardiography data.

	HR (bpm)	PQ (ms)	QRS (ms)
**WT**	445.5	17.40	8.59
***STIM+/-***	454.7	17.84	8.55
***p***	0.88	0.91	0.54

Electrocardiogram with concurrent M-mode echocardiography were done in *STIM1*^+/–^and WT mice at 10 weeks of age anesthetized with isoflurane (1%). Electrocardiogram including HR, PQ interval, QRS interval are shown. Statistical data are presented as means ± SEM.

**Table 3:** Echocardiography data.

	ivs (mm)	pw (mm)	Dd (mm)	Ds (mm)	EF (%)	FS (%)	LVVd (μl)	LVVs (μl)	SV (μl)	LV Mass (mg)
**WT**	0.8	0.81	3.52	2.35	64.1	35	54.9	21.6	31.4	103.9
***STIM+/-***	0.81	0.81	3.52	2.36	63.6	34.6	55.2	23.7	32.6	105.1
***p***	0.82	0.91	0.96	0.94	0.88	0.88	0.96	0.63	0.67	0.9

Echocardiographic characterizations including interventricular septum wall thickness (ivs), posterior wall thickness (pw), end-diastolic left ventricular diameter (Dd), end-systolic left ventricular diameter (Ds), ejection fraction (EF), fractional shortening percentage (FS), end-diastolic left ventricular volume (LVVd), end-systolic left ventricular volume (LVVs), stroke volume (SV), and estimated left ventricular mass (LV Mass) are shown. Statistical data are presented as means ± SEM

### 3. Cardiac hypertrophy in response to aortic constriction

**Fig 2:**
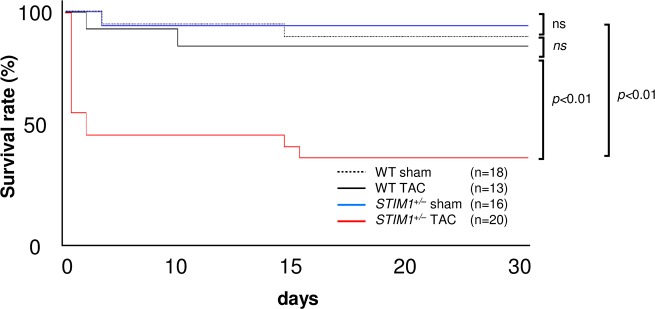
Survival rate of mice. Survival rate of *STIM1*^*+/–*^and WT mice after transverse aortic constriction (TAC) or sham surgery, as analyzed by the Kaplan-Meier method and log-rank test.

To elucidate the role of STIM1 in cardiac hypertrophy, *STIM1*^+/–^mice were subjected to pressure overload with a TAC procedure at 10 weeks of age. *STIM1*^+/–^and WT littermates were divided into 4 groups: WT sham (n = 18), WT TAC (n = 13), *STIM1*^+/–^sham (n = 16), and *STIM1*^+/–^TAC (n = 20). To investigate mortality, the survival rates after TAC surgery were analyzed using the Kaplan-Meier method and log-rank test. Surprisingly, more than 40% of *STIM1*^+/–^TAC mice died within 48 h of surgery (Fig 2). The autopsies of *STIM1*^+/–^TAC mice that died shortly after surgery showed no remarkable perivascular edema in the lungs or ventricular dilation indicative of heart failure. The survival rate after 30 days was ~35%. These findings imply that the cause of acute death was lethal arrhythmia.

**Fig 3:**
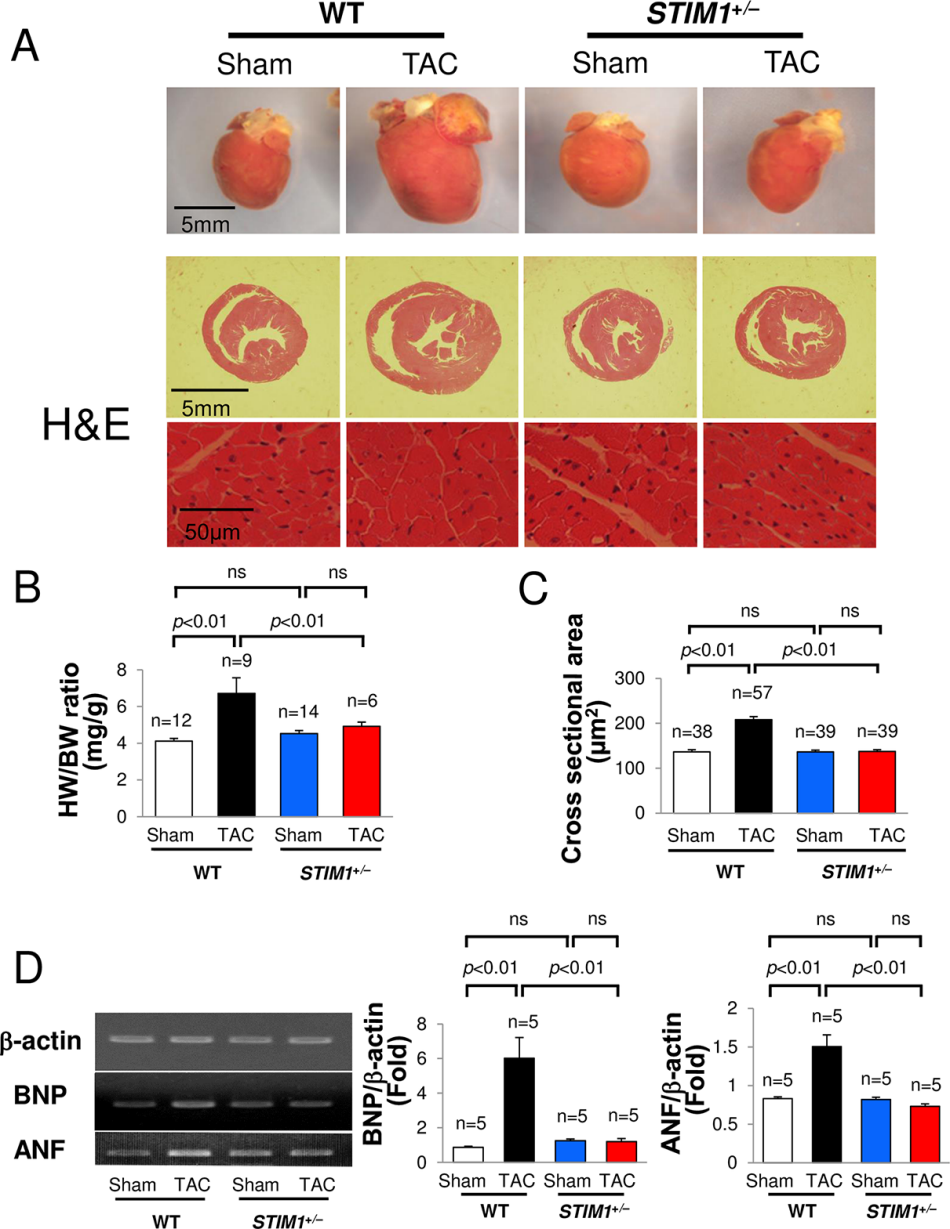
Cardiac hypertrophy in response to TAC. (A) Photographs and histology of hearts from *STIM1*^+/–^and WT male mice 4 weeks after TAC or sham surgery. Hearts were sectioned horizontally and hematoxylin & eosin (H&E) staining was performed. Cardiac hypertrophy was present in the hearts of WT TAC mice. (B) Heart weight (HW) to body weight (BW) ratio (mg/g). (C) Cross sectional area (**μ**m^2^). (D) Comparative RT-PCR analysis of gene expression for brain natriuretic protein (BNP), atrial natriuretic protein (ANF), and β-actin. Each experiment was repeated 5 times with the same result. All values are means ± SEM.

Four weeks after the TAC or sham operation, mice were sacrificed and their hearts were isolated. The banded portion of the aorta was observed via stereomicroscope and the constriction rate was calculated. Greater than 94% constriction was considered a successful TAC operation. The hearts of WT TAC mice had remarkable cardiac hypertrophy, while hearts from *STIM1*^+/–^TAC mice showed no marked changes (Fig 3A). A greater than 1.5-fold increase in the HW/BW ratio and cross-sectional area of myocytes within the LV wall was observed in WT TAC mice compared with WT sham mice (Fig 3B and 3C). *STIM1*^+/–^mice had significantly less cardiac hypertrophy than WT TAC mice. These results suggest that *STIM1*^+/–^mice failed to manifest the appearance of cardiac hypertrophy.

Comparative RT-PCR analyses were performed on the LVs of *STIM1*^+/–^and WT mice 4 weeks after TAC or sham surgery. We examined the expression of BNP and ANF, cardiac fetal genes used as markers of cardiac hypertrophy. The RT-PCR analysis revealed increased levels of BNP and ANF in the WT TAC group compared with those in the WT sham group (Fig 3D, lane 2). In contrast, increases in BNP and ANF in *STIM1*^+/–^TAC mice were attenuated (Fig 3D, lane 4) compared with those in WT TAC mice. The RT-PCR analysis revealed comparable amplification of β-actin, suggesting equivalent experimental conditions. The experiment was repeated in 5 independent hearts with similar results; the statistical data are presented. These data suggest that *STIM1*^+/–^TAC mice have a maladaptive response to pressure overload.

### 4. Expression of TRPC subtypes in STIM1^+/–^hearts

**Fig 4:**
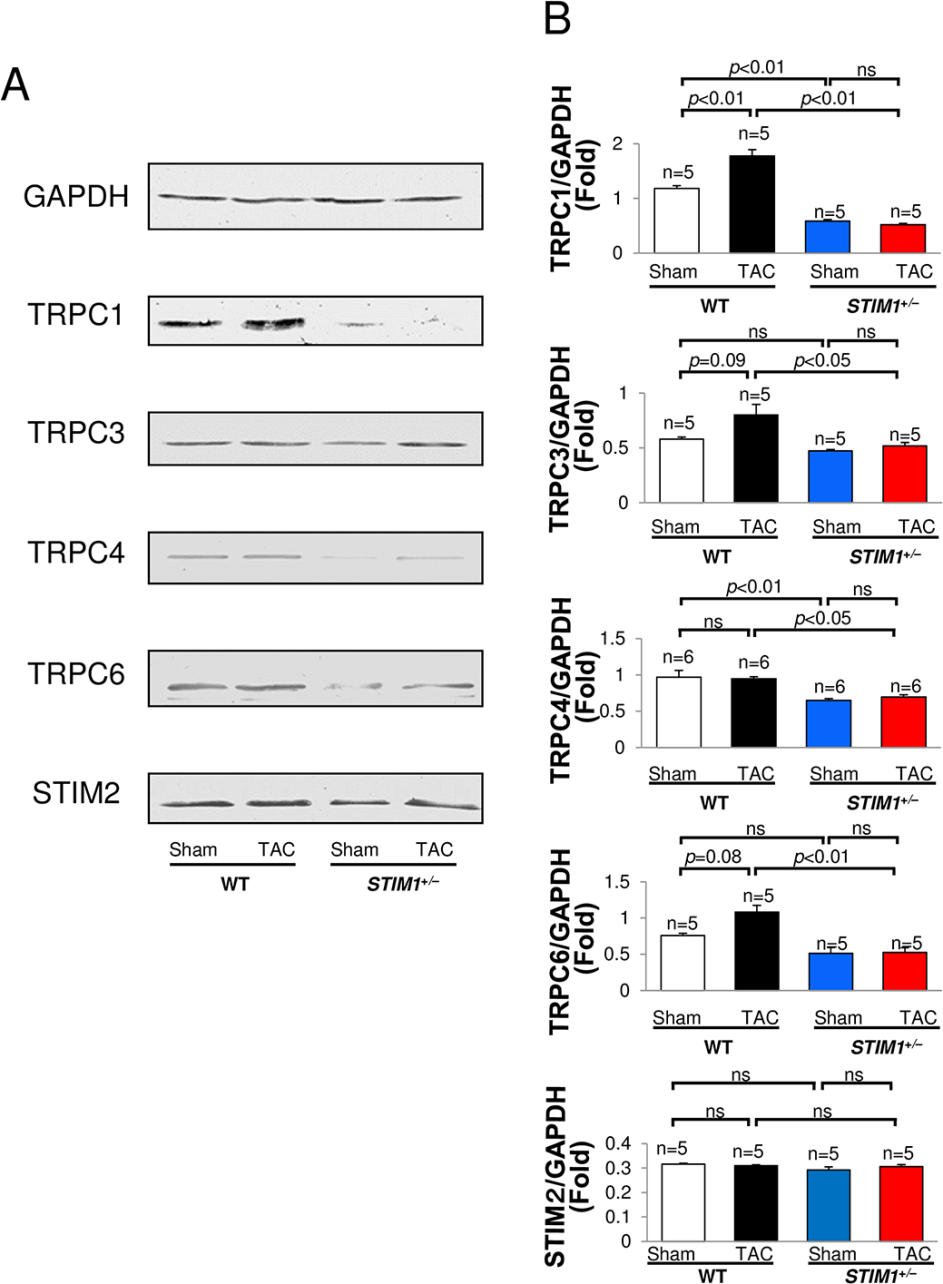
Immunoblotting for TRPCs, STIM1 and STIM2. Immunoblotting for transient receptor potential channels (TRPCs), STIM1 and STIM2 subtype in hearts from *STIM1*^+/–^and WT mice after TAC. Each experiment was repeated 5 or more times with the same results. All values are means ± SEM.

Upregulation of TRPCs was implicated in the development of cardiac hypertrophy [1–6]. TRPC1, TRPC3, and TRPC6 have conserved NFAT consensus sequences in their promoters [2, 6, 10, 28]. Once activated, TRPC-mediated Ca^2+^ entry presumably activates NFAT and facilitates the expression of other TRPCs. In the present study, Western blot analysis revealed constitutive expression of TRPCs in the hearts of adult mice (Fig 4). Consistent with our previous studies [4–7], TRPC1 expression was significantly increased in the hearts of WT TAC mice compared with WT sham mice (151 ± 11%, n = 5). As for TRPC3 and TRPC6, their expression tended to be increased in WT TAC mice, as previously reported [1, 2, 29], though it was not statistically significant. On the other hand, *STIM1*^+/–^mice had very low levels of TRPCs (TRPC1, TRPC3, TRPC4 and TRPC6) in the sham group, and upregulation of TRPCs was not observed in TAC-operated hearts. When compared between *STIM1*^+/–^and WT TAC mice, the expression level of TRPC1, TRPC3, TRPC4 and TRPC6 was significantly lower in *STIM1*^+/–^than WT TAC mice hearts. Anti-GAPDH antibodies were used to verify that comparable amounts of protein were loaded in each lane of the gel; each experiment was repeated 5 times with the same results. These data suggest that STIM1 deficiency attenuates upregulation of TRPCs.

### 5. NFATc4 in STIM1^+/–^hearts

**Fig 5:**
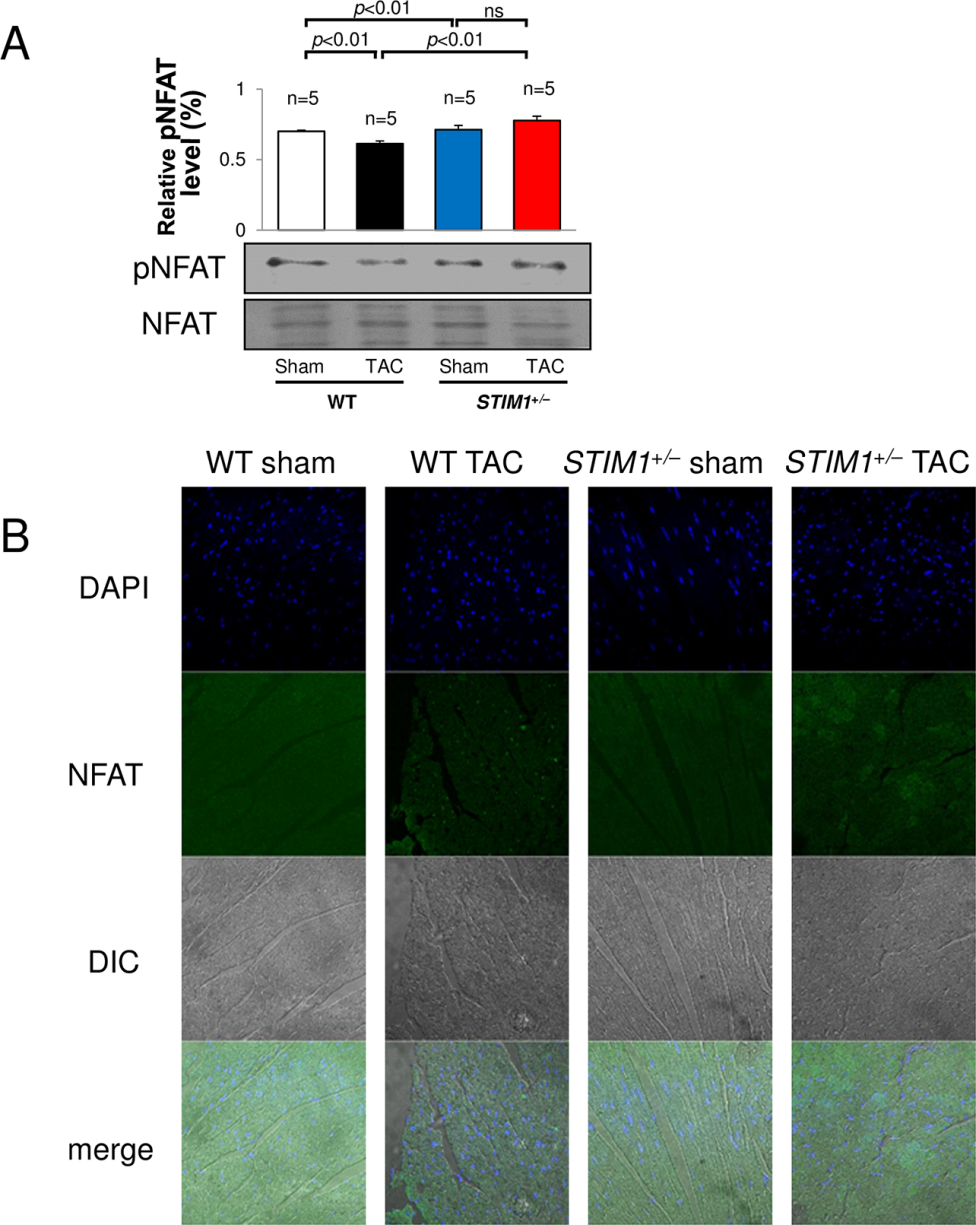
NFAT activation. Immunoblotting (A) and immunostaining (B) for the NFAT and the phosphorylated NFATc4 (pNFAT) in hearts from *STIM1*^+/–^and WT mice after TAC. Each experiment was repeated 5 or more times with the same results. All values are means ± SEM.

To examine subsequent NFAT signaling as a downstream event of Ca^2+^ related pathway in cardiac hypertrophy, we evaluated the NFAT activity by using Western blot and immunostaining (Fig 5). The relative pNFAT level was significantly decreased in the hearts of WT TAC compared with WT sham (88 ± 3.3%, n = 5). However, *STIM1*^+/–^mice had stationary level of relative pNFAT in both sham and TAC compared with WT. In Fig 5B, the NFAT proteins appeared green under confocal laser scanning microscope. Almost NFAT was accumulated in the nucleus of cardiomyocytes of WT TAC. These results suggest that NFAT activity is related to the development of cardiac hypertrophy and the up-regulation of TRPC1.

## Discussion

This study provides novel insights into the role of STIM1 in the heart. First, the morphological and functional examinations showed that STIM1 haploinsufficiency (STIM1 protein levels decreased to 27% of WT) exerted no influence on the basal cardiac phenotype in mice up to at least 10 weeks of age. Second, TAC-treated *STIM1*^*+/–*^mice failed to manifest evidence of cardiac hypertrophy. Third, *STIM1*^*+/–*^mice were more susceptible to TAC-induced mortality. Collectively, these data suggest that STIM1 haploinsufficiency does not influence the basal cardiac phenotype up to 10 weeks of age, but confers a maladaptation, which renders it incapable of hypertrophic growth under pressure-overload conditions.

### Basal cardiac phenotype of *STIM1*^*+/–*^mice

In skeletal muscle, Ca^2+^ signaling governed by STIM1 has been reported to play a central role in muscle growth and differentiation [30]. In fact, patients with STIM1 deficiency suffered from nonprogressive myopathy and immunodeficiency [15], and those with constitutive activation of STIM1 exhibited tubular-aggregate myopathy [16]. On the other hand, functional role of STIM1 seems minimal in the normal heart during development. In the present study, the protein level of STIM1 was reduced to 27% in *STIM1*^+/–^mice, and there were no apparent morphologic or histological differences between the heart of *STIM1*^+/–^and WT mice at 10 weeks of age. Furthermore, hemodynamic echo and ECG parameters were also unremarkable in *STIM1*^+/–^. These findings are in good agreement with a previous study using heart-specific STIM1-KO mice in which STIM1 expression was reduced by 92% [31]. There were no gross morphological or functional differences between heart-specific STIM1-KO and control hearts up to 20 weeks of age, and thereafter cardiac function started to decline and progressively worsened by 36 weeks, accompanied by significant alterations in mitochondrial morphology. These authors suggested that STIM1 is likely unnecessary for normal cardiac development and function, but it plays an essential role in normal cardiac function in the adult heart. In humans, autosomal-recessive mutations in the STIM1 result in combined immunodeficiency, immunodysregulation, ectodermal dysplasia, and nonprogressive myopathy [15]. The prognosis of these patients is poor and most of the patients died in childhood with fatal consequences mainly due to immune response failure. Thus, it is not yet fully examined whether or not the cardiac function is impaired in the adult patients. Nevertheless, all these findings support a view that STIM1 has little effect on normal cardiac contraction and electrical conduction system in mice up to at least 10 weeks of age.

### Maladaptive response to pressure overload in *STIM1*^*+/–*^mice

In contrast to WT, *STIM1*^*+/–*^mice showed no evidence of cardiac hypertrophy, such as growth of cardiomyocytes and fetal gene expression, when subjected to TAC-induced hemodynamic stress. In addition, the upregulation of TRPC1 was inhibited in the hearts of *STIM1*^*+/–*^mice, and the expression of TRPC1, TRPC3, TRPC4 and TRPC6 was significantly lower in *STIM1*^*+/–*^than in WT TAC mice. Several studies have shown interaction between STIM1 and TRPCs. In particular, TRPC1, TRPC3 and TRPC6 have been found to be upregulated in response to pressure overload and a model of calcineurin-mediated cardiomyopathy [1–7]. Furthermore, endothelin-1 treatment of cultured rat myocytes enhanced TRPC1 expression, SOCE, and NFAT activation, and the knockdown of STIM1 suppressed these effects, thereby preventing a hypertrophic response [7]. Taken together, we may safely conclude that STIM1 haploinsufficiency decreases TRPCs expression, and that the expression of TRPCs does not reach the necessary level for cardiac hypertrophy during pressure overload.

In lymphocyte SOCE signaling, STIM2 compensation for STIM1 has been reported. However, STIM2 did not compensate for STIM1 haploinsufficiency in our pressure-overload model. These results suggest that the constitutive expression level of STIM1 is a critical factor in the development of cardiac hypertrophy, and raise the possibility that STIM1-deficient patients have an anti-hypertrophic phenotype. The cardiac hypertrophic response is a compensatory reaction against an increased load, but if the adaptive response progresses beyond a certain point, various cardiovascular events, such as heart failure, can accompany cardiac hypertrophy. Therefore, these data suggest that STIM1 inhibition might be a promising approach for cardioprotection in hypertrophied hearts.

Indeed, Hulot et al. showed that *Stim-1* gene silencing by viral gene transfer protected rats from pressure overload-induced cardiac hypertrophy [9]. Latest report showed that *Stim1* silencing prevents the development of pressure overload–induced hypertrophy in mice by using in vivo gene delivery of specific short hairpin RNAs [32]. They showed that mice started to present a progressive cardiac dilation and dysfunction with reduced cardiac STIM1 expression. On the other hand, Correll et al. showed that transgenic mice with STIM1 overexpression exhibited sudden cardiac death as early as 6 weeks of age, while mice surviving past 12 weeks of age developed heart failure with hypertrophy, induction of the fetal gene program, histopathology and mitochondrial structural alterations, loss of ventricular functional performance and pulmonary edema [33]. These reports provided evidences that reduced STIM1 leaded to the protection against cardiac hypertrophy, though the overexpression of STIM1exerted an adverse effect. However, in this study, *STIM1*^+/–^mice were less tolerant of increased afterload, as >30% of *STIM1*^+/–^TAC mice died within 48 h of surgery (Fig 3A). Considering the low mortality of WT TAC and *STIM1*^+/–^sham mice, it is likely that the high mortality after TAC can be attributed to the inability of *STIM1*^+/–^mice to adapt to pressure overload. This view is supported by a previous study in which STIM1 KO mice had a limited life expectancy even in the absence of cardiac stress [28]. Collins et al. showed there was no effect of STIM1 loss on cardiac function up to 20 weeks though after 20 weeks there were significant impairments in function, with signs of ER stress as early as 12 weeks. At present, it is consistent in that STIM1 is a multifunctional regulator of cardiac myocytes. Moreover, STIM1 was identified as a strong candidate gene responsible for the exaggerated sympathetic response to stress [34]. Hence, STIM1 haploinsufficiency might exhibit a maladaptive response to environmental stressors including pressure overload. These findings may limit a STIM1-repression treatment approach for heart disease. Although modifying the expression of STIM1 may be a promising therapeutic approach for cardiac hypertrophy and heart failure, identifying the point of transition from an adaptive state to a decompensated response and making a timely inhibition of STIM1 might also be of great importance.

The early death observed in *STIM1*^+/–^TAC is striking in the present study. Although we were technically correct in attributing this to an inability to adapt to pressure overload, possible causes of the early death could not be clarified in the present study. The impairment of hypertrophy in the surviving mice is noteworthy, but it is impossible to conclude that such impairment contributed to the early death, since it was too rapid for hypertrophy to be a significant factor. Furthermore, the autopsies of *STIM1*^+/–^TAC mice that died shortly after surgery showed no remarkable perivascular edema in the lungs or ventricular dilation indicative of heart failure. We speculate that these animals might have died acutely due to lethal arrhythmias, which would fit to the role of STIM1 as a Ca^2+^ sensor, but this was not examined, and no ECG or echo data were obtained within 48 hours after TAC. Further experiments are clearly necessary to elucidate this point. On the other hand, the slope of the Kaplan-Meier curve after the initial 48 hours post-TAC was similar between *STIM1*^+/–^and WT TAC group. This suggests that the inability to undergo hypertrophy is not a key factor in the subsequent death of *STIM1*^+/–^-TAC mice. Also, the rapid death followed by modest rate of death after TAC may imply that there is an incomplete penetrance of phenotype in the STIM1 heterozygotes. Namely, the sickest animals in which the STIM1 expression might have been more suppressed markedly died in the initial 48 hours, while hardier animals with modest suppression of STIM1 expression survived to the study endpoint. At present, we have no clear answer regarding this important issue. In any case, however, it seems difficult to associate the impairment of hypertrophy with the early death observed.

### Conclusions

The hearts of STIM1 haploinsufficient mice had a superficial resemblance to the WT phenotype. However, STIM1 haploinsufficient mice displayed a maladaptive response to cardiac overload.

## Supporting information

S1 Table Primer sequences. RT-PCR was performed using each primer of stromal interaction molecule (STIM) 1, STIM2, atrial natriuretic protein (ANF), brain natriuretic protein (BNP) and β-actin. Primer sequences are provided.
(PPT)

## References

[1] Onohara N, Nishida M, Inoue R, Kobayashi H, Sumimoto H, Sato Y, et al TRPC3 and TRPC6 are essential for angiotensin II-induced cardiac hypertrophy. EMBO J. 2006 11 15;25(22):5305–16. doi: 10.1038/sj.emboj.7601417 17082763 10.1038/sj.emboj.7601417 PMC1636614

[2] Kuwahara K, Wang Y, McAnally J, Richardson JA, Bassel-Duby R, Hill JA, et al TRPC6 fulfills a calcineurin signaling circuit during pathologic cardiac remodeling. J. Clin. Invest. 2006 12;116(12):3114–26. doi: 10.1172/JCI27702 17099778 10.1172/JCI27702 PMC1635163

[3] Seth M, Zhang ZS, Mao L, Graham V, Burch J, Stiber J, et al TRPC1 channels are critical for hypertrophic signaling in the heart. Circ Res. 2009 11 6;105(10):1023–30 doi: 10.1161/CIRCRESAHA.109.206581 19797170 10.1161/CIRCRESAHA.109.206581 PMC2881555

[4] Ohba T, Watanabe H, Takahashi Y, Suzuki T, Miyoshi I, Nakayama S, et al Regulatory role of neuron-restrictive silencing factor in expression of TRPC1. Biochem Biophys Res Commun. 2006 12 22;351(3):764–70. doi: 10.1016/j.bbrc.2006.10.107 17084381 10.1016/j.bbrc.2006.10.107

[5] Ohba T, Watanabe H, Murakami M, Takahashi Y, Ito H. Increased expression of transient receptor potential c1 in rats with hypertensive hypertrophy. Akita J Med. 2005 3 31;32(3–4):201–7.

[6] Ohba T, Watanabe H, Murakami M, Takahashi Y, Iino K, Kuromitsu S, et al Up-regulation of TRPC1 in the development of cardiac hypertrophy. J Mol Cell Cardiol. 2007 3;42(3):498–507. doi: 10.1016/j.yjmcc.2006.10.020 17174323 10.1016/j.yjmcc.2006.10.020

[7] Ohba T, Watanabe H, Murakami M, Sato T, Ono K, Ito H. Essential role of STIM1 in the development of cardiomyocyte hypertrophy. Biochem Biophys Res Commun. 2009 11 6;389(1):172–6. doi: 10.1016/j.bbrc.2009.08.117 19715666 10.1016/j.bbrc.2009.08.117

[8] Voelkers M, Salz M, Herzog N, Frank D, Dolatabadi N, Frey N, et al Orai1 and Stim1 regulate normal and hypertrophic growth in cardiomyocytes. J Mol Cell Cardiol. 2010 6;48(6):1329–34. doi: 10.1016/j.yjmcc.2010.01.020 20138887 10.1016/j.yjmcc.2010.01.020 PMC5511312

[9] Hulot JS, Fauconnier J, Ramanujam D, Chaanine A, Aubart F, Sassi Y, et al Critical role for stromal interaction molecule 1 in cardiac hypertrophy. Circulation. 2011 8 16;124(7):796–805. doi: 10.1161/CIRCULATIONAHA.111.031229 21810664 10.1161/CIRCULATIONAHA.111.031229 PMC3428713

[10] Luo X, Hojayev B, Jiang N, Wang ZV, Tandan S, Rakalin A, et al STIM1-dependent store-operated Ca^2+^ entry is required for pathological cardiac hypertrophy. J Mol Cell Cardiol. 2012 1;52(1):136–47. doi: 10.1016/j.yjmcc.2011.11.003 22108056 10.1016/j.yjmcc.2011.11.003 PMC3247164

[11] Kar P, Parekh A. STIM proteins, Orai1 and gene expression. Channels. 2013 Sep-Oct;7(5):374–8 doi: 10.4161/chan.25298 23765192 10.4161/chan.25298 PMC3913760

[12] Zhou MH, Zheng H, Si H, Jin Y, Peng JM, He L, et al Stromal interaction molecule 1 (STIM1) and Orai1 mediate histamine-evoked calcium entry and nuclear factor of activated T-cells (NFAT) signaling in human umbilical vein endothelial cells.J Biol Chem. 2014 10 17;289(42):29446–56. doi: 10.1074/jbc.M114.578492 25190815 10.1074/jbc.M114.578492 PMC4200292

[13] Ambudkar IS, de Souza LB, Ong HL. TRPC1, Orai1, and STIM1 in SOCE: Friends in tight spaces. Cell Calcium. 2017 5;63:33–39. doi: 10.1016/j.ceca.2016.12.009 28089266 10.1016/j.ceca.2016.12.009 PMC5466534

[14] Oh-Hora M, Yamashita M, Hogan PG, Sharma S, Lamperti E, Chung W, et al Dual functions for the endoplasmic reticulum calcium sensors STIM1 and STIM2 in T cell activation and tolerance. Nat Immunol. 2008 4;9(4):432–43. doi: 10.1038/ni1574 18327260 10.1038/ni1574 PMC2737533

[15] Picard C, McCarl CA, Papolos A, Khalil S, Lüthy K, Hivroz C, et al STIM1 mutation associated with a syndrome of immunodeficiency and autoimmunity. N Engl J Med. 2009 5 7;360(19):1971–80. doi: 10.1056/NEJMoa0900082 19420366 10.1056/NEJMoa0900082 PMC2851618

[16] Böhm J, Chevessier F, Maues De Paula A, Attarian S, Feger C, Hantaï D, et al Constitutive activation of the calcium sensor STIM1 causes tubular-aggregate myopathy. Am J Hum Genet. 2013 2 7;92(2):271–8. doi: 10.1016/j.ajhg.2012.12.007 23332920 10.1016/j.ajhg.2012.12.007 PMC3567276

[17] Fuchs S, Rensing-Ehl A, Speckmann C, Bengsch B, Schmitt-Graeff A, Bondzio I, et al Antiviral and regulatory T cell immunity in a patient with stromal interaction molecule 1 deficiency. J Immunol. 2012 2 1;188(3):1523–33. doi: 10.4049/jimmunol.1102507 22190180 10.4049/jimmunol.1102507 PMC3262903

[18] Wang S, Choi M, Richardson AS, Reid BM, Seymen F, Yildirim M, et al STIM1 and SLC24A4 Are Critical for Enamel Maturation. J Dent Res. 2014 7;93(7 Suppl):94S–100S. 24621671 10.1177/0022034514527971 PMC4107542

[19] Schaballie H, Rodriguez R, Martin E, Moens L, Frans G, Lenoir C, et al A novel hypomorphic mutation in STIM1 results in a late-onset immunodeficiency. J Allergy Clin Immunol. 2015 9;136(3):816–819. doi: 10.1016/j.jaci.2015.03.009 25935105 10.1016/j.jaci.2015.03.009

[20] Baba Y, Nishida K, Fujii Y, Hirano T, Hikida M, Kurosaki T. Essential function for the calcium sensor STIM1 in mast cell activation and anaphylactic responses. Nat Immunol. 2008 1;9(1):81–8. doi: 10.1038/ni1546 18059272 10.1038/ni1546

[21] Kassiri Z, Oudit GY, Sanchez O, Dawood F, Mohammed FF, Nuttall RK, et al Combination of tumor necrosis factor-alpha ablation and matrix metalloproteinase inhibition prevents heart failure after pressure overload in tissue inhibitor of metalloproteinase-3 knock-out mice. Circ Res. 2005 8 19;97(4):380–90. doi: 10.1161/01.RES.0000178789.16929.cf 16037568 10.1161/01.RES.0000178789.16929.cf

[22] Rockman HA, Ross RS, Harris AN, Knowlton KU, Steinhelper ME, Field LJ, et al Segregation of atrial-specific and inducible expression of an atrial natriuretic factor transgene in an in vivo murine model of cardiac hypertrophy. Proc Natl Acad Sci U S A. 1991 9 15;88(18):8277–81. 1832775 10.1073/pnas.88.18.8277 PMC52490

[23] Hu P, Zhang D, Swenson L, Chakrabarti G, Abel ED, Litwin SE. Minimally invasive aortic banding in mice: effects of altered cardiomyocyte insulin signaling during pressure overload. Am J Physiol Heart Circ Physiol. 2003 9;285(3):H1261–9. doi: 10.1152/ajpheart.00108.2003 12738623 10.1152/ajpheart.00108.2003

[24] Bradshaw AD, Baicu CF, Rentz TJ, Van Laer AO, Boggs J, Lacy JM, Zile MR. Pressure overload-induced alterations in fibrillar collagen content and myocardial diastolic function: role of secreted protein acidic and rich in cysteine (SPARC) in post-synthetic procollagen processing. Circulation. 2009 1 20;119(2):269–80. doi: 10.1161/CIRCULATIONAHA.108.773424 19118257 10.1161/CIRCULATIONAHA.108.773424 PMC2734276

[25] Faerber G, Barreto-Perreia F, Schoepe M, Gilsbach R, Schrepper A, Schwarzer M, et al Induction of heart failure by minimally invasive aortic constriction in mice: reduced peroxisome proliferator-activated receptor γ coactivator levels and mitochondrial dysfunction. J Thorac Cardiovasc Surg. 2011 2;141(2):492–500. doi: 10.1016/j.jtcvs.2010.03.029 20447656 10.1016/j.jtcvs.2010.03.029

[26] Stiber J, Hawkins A, Zhang ZS, Wang S, Burch J, Graham V, et al STIM1 signalling controls store-operated calcium entry required for development and contractile function in skeletal muscle. Nat Cell Biol. 2008 6;10(6):688–97. doi: 10.1038/ncb1731 18488020 10.1038/ncb1731 PMC2694045

[27] Ritchie MF, Yue C, Zhou Y, Houghton PJ, Soboloff J. Wilms tumor suppressor 1 (WT1) and early growth response 1 (EGR1) are regulators of STIM1 expression. J Biol Chem. 2010 4 2;285(14):10591–6. doi: 10.1074/jbc.M109.083493 20123987 10.1074/jbc.M109.083493 PMC2856267

[28] Paria BC, Malik AB, Kwiatek AM, Rahman A, May MJ, Ghosh S, et al Tumor necrosis factor-alpha induces nuclear factor-kappaB-dependent TRPC1 expression in endothelial cells. J Biol Chem. 2003 9 26;278(39):37195–203. doi: 10.1074/jbc.M304287200 12855710 10.1074/jbc.M304287200

[29] Bush EW, Hood DB, Papst PJ, Chapo JA, Minobe W, Bristow MR, et al Canonical transient receptor potential channels promote cardiomyocyte hypertrophy through activation of calcineurin signaling. J Biol Chem. 2006 11 3;281(44):33487–96. doi: 10.1074/jbc.M605536200 16950785 10.1074/jbc.M605536200

[30] Li T, Finch EA, Graham V, Zhang ZS, Ding JD, Burch J, et al STIM1-Ca(2+) signaling is required for the hypertrophic growth of skeletal muscle in mice. Mol Cell Biol. 2012 8;32(15):3009–17. doi: 10.1128/MCB.06599-11 22645307 10.1128/MCB.06599-11 PMC3434510

[31] Collins HE, He L, Zou L, Qu J, Zhou L, Litovsky SH, et al Stromal interaction molecule 1 is essential for normal cardiac homeostasis through modulation of ER and mitochondrial function. Am J Physiol Heart Circ Physiol. 2014 4 15;306(8):H1231–9. doi: 10.1152/ajpheart.00075.2014 24585777 10.1152/ajpheart.00075.2014 PMC3989749

[32] Bénard L, Oh JG, Cacheux M, Lee A, Nonnenmacher M, Matasic DS, et al Cardiac Stim1 Silencing Impairs Adaptive Hypertrophy and Promotes Heart Failure Through Inactivation of mTORC2/Akt Signaling. Circulation. 2016 4 12;133(15):1458–71. doi: 10.1161/CIRCULATIONAHA.115.020678 26936863 10.1161/CIRCULATIONAHA.115.020678 PMC4829441

[33] Correll RN, Goonasekera SA, van Berlo JH, Burr AR, Accornero F, Zhang H, et al STIM1 elevation in the heart results in aberrant Ca^2+^ handling and cardiomyopathy. J Mol Cell Cardiol. 2015 10;87:38–47. doi: 10.1016/j.yjmcc.2015.07.032 26241845 10.1016/j.yjmcc.2015.07.032 PMC4637225

[34] Ferdaus MZ, Xiao B, Ohara H, Nemoto K, Harada Y, Saar K, et al Identification of Stim1 as a candidate gene for exaggerated sympathetic response to stress in the stroke-prone spontaneously hypertensive rat. PLoS One. 2014 4 15;9(4):e95091 doi: 10.1371/journal.pone.0095091 24736434 10.1371/journal.pone.0095091 PMC3988177

