# Supplementary material for: Stromal interaction molecule 1 haploinsufficiency causes maladaptive response to pressure overload
Source: PLoS One. 2017 Nov 16;12(11):e0187950. doi: 10.1371/journal.pone.0187950 (PMC5690472; doi:10.1371/journal.pone.0187950)
Supplement: S1 Table — RT-PCR was performed using each primer of stromal interaction molecule (STIM) 1, STIM2, atrial natriuretic protein (ANF), brain natriuretic protein (BNP) and β-actin. Primer sequences are provided. (PPT) [file pone.0187950.s001.ppt]

## Slide 1
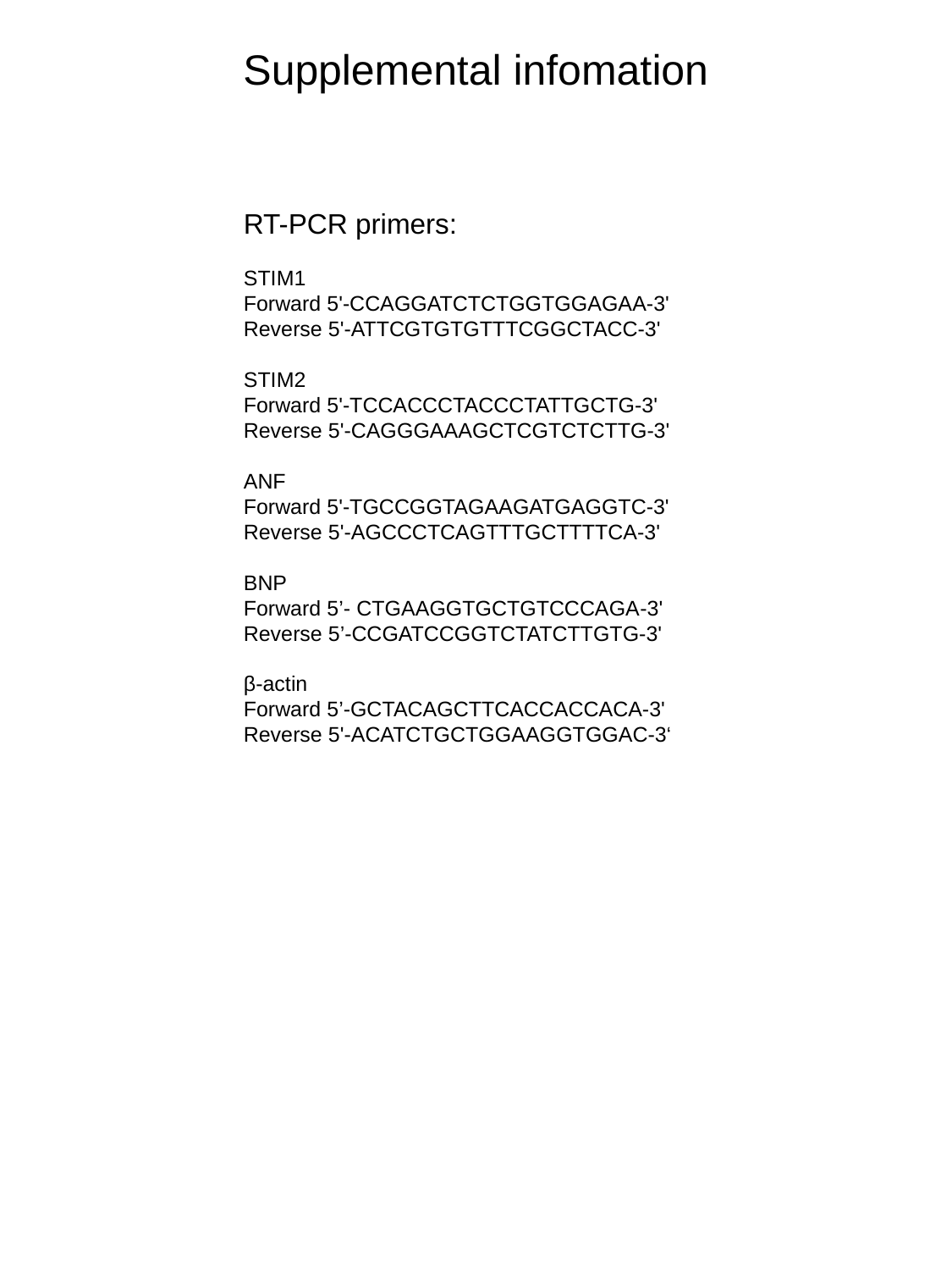

Supplemental infomation
RT-PCR primers:
STIM1
Forward 5'‑CCAGGATCTCTGGTGGAGAA‑3'
Reverse 5'‑ATTCGTGTGTTTCGGCTACC‑3'
STIM2
Forward 5'‑TCCACCCTACCCTATTGCTG‑3'
Reverse 5'‑CAGGGAAAGCTCGTCTCTTG‑3'
ANF
Forward 5'‑TGCCGGTAGAAGATGAGGTC‑3'
Reverse 5'‑AGCCCTCAGTTTGCTTTTCA‑3'
BNP
Forward 5’- CTGAAGGTGCTGTCCCAGA‑3'
Reverse 5’-CCGATCCGGTCTATCTTGTG‑3'
β-actin
Forward 5’‑GCTACAGCTTCACCACCACA‑3'
Reverse 5'‑ACATCTGCTGGAAGGTGGAC‑3‘
